# Nephrologists’ perception of the French national guidelines in nephrology

**DOI:** 10.1038/s41598-025-18712-5

**Published:** 2025-09-29

**Authors:** Latame Komla Adoli, Cécile Vigneau, Arnaud Campeon, Cécile Couchoud, Valérie Chatelet, Thierry Lobbedez, Eric Daugas, Florian Bayer, Elsa Vabret, Jean-Philippe Jais, Sahar Bayat-Makoei

**Affiliations:** 1 Univ Rennes, Ehesp, Cnrs, Inserm, Arènes - Umr 6051, Rsms - U1309, 35000 Rennes, France; 2 Univ Rennes, Chu Rennes, Inserm, Ehesp, Irset (Institut de Recherche en Santé, Environnement Et Travail) - Umr_s 1085, 35000 Rennes, France; 3 Arènes-UMR 6051, ISSAV, EHESP, CNRS, 35000 Rennes, France; 4 Renal Epidemiology and Information Network (REIN) Registry, Biomedecine Agency, Saint-Denis-La-Plaine, France; 5 U1086 Inserm, Anticipe, Centre De Lutte Contre Le Cancer François Baclesse, Centre Universitaire Des Maladies Rénales, Caen, France; 6 Inserm U1149 Université Paris Cité, Assistance Publique-Hôpitaux de Paris, Service De Néphrologie, Hôpital Bichat, Paris, France; 7 Biostatistics Unit, Necker-Enfants Malades Hospital, AP-HP, Imagine Institute, Université Paris-Cité, Paris, France

**Keywords:** Guidelines, Nephrology, Perception, Mixed methods, Nephrology, Health policy

## Abstract

In France, the “Haute Autorité de Santé” (HAS), an independent public scientific authority, regularly publishes guidelines targeted to healthcare professionals. As their implementation is left to the healthcare professionals’ discretion, their perception could influence their application. The aim of this study was to assess the nephrologists’ perception of the HAS guidelines on nephrology in general and on the access to the kidney transplant waiting list. We used a mixed methods approach with an exploratory design combining analysis of qualitative and quantitative data. Nephrologists practicing in France were included. We collected qualitative data in face-to-face semi-structured interviews and identified the main themes through an inductive thematic analysis. We collected quantitative data through an online questionnaire designed based on the qualitative findings. The analysis of interviews with 45 nephrologists (22 women) identified three main themes: (i) nephrologists’ knowledge and sources of information on HAS guidelines in nephrology; (ii) their perception of these guidelines and relevance to medical practice; (iii) age limit to access the kidney transplant waiting list in the 2015 guidelines. The quantitative analysis included 126 nephrologists (47.6% women), among whom 84.1% had already heard about these guidelines. Respectively, 85.8% and 80.2% found the guidelines “clear” and “complete”. Overall, the quantitative data confirmed the qualitative findings. This study shows how nephrologists perceive the HAS guidelines. This will inform discussions on how to make these guidelines more accessible to nephrologists. New studies could be carried out to better quantify and qualify the effect of these guidelines

## Introduction

The management of chronic kidney disease (CKD) involves a succession of stages: etiology treatment, kidney function preservation, preparation for dialysis, registration on the waiting list and transplantation for eligible patients, ideally before dialysis. The increasing prevalence of kidney disease, therapeutic constraints, and barriers to kidney transplantation access are just some of the difficulties hampering the CKD care trajectory^1,2^. Although the diagnosis is sometimes made at an advanced stage, thus necessitating the urgent initiation of dialysis^3,4^, significant progress has been made in CKD management before the renal replacement therapy stage^5^. The application of guidelines represents an essential lever to optimize the care trajectory and management of patients with CKD^6^.

The introduction of guidelines in healthcare goes back several years^7^. In nephrology, the Kidney Disease Improving Global Outcomes (KDIGO) group regularly proposes guidelines to improve the quality of patient care^8,9^. In France, the Haute Autorité de Santé (HAS), an independent scientific public authority, regularly publishes guidelines in the healthcare, social and medico-social fields that are made available to healthcare professionals^10,11^. In 2012, HAS published guidelines on the CKD care trajectory in adult patients targeted to the healthcare professionals involved in their management, with the aim of describing the entire care trajectory for patients with CKD. For instance, they include recommendations on the number and sequence of consultations with different healthcare professionals. These guidelines were updated in 2021 and 2023. In addition, in 2015, a guideline on kidney transplantation was published to harmonize professional practices for registering patients on the transplant waiting list among regions, and thus reducing disparities^12^. In this text, the age limit beyond which non-referral of patients to a transplant team is justified was set at 85 years. Yet, disparities persist in the patients’ access to the waiting list and to transplantation. A study on patients who started dialysis showed that women older than 60 years have lower access to the waiting list and to kidney transplantation^13^. As the application of the HAS guidelines is left to the healthcare professionals’ discretion, their implementation could be influenced by how the nephrologists’ perceive them. The aim of this study was to describe the nephrologists’ perception of the HAS guidelines in nephrology in general and particularly on the access to the renal transplant waiting list.

## Methods

This work is part of a research project to study women access to kidney transplantation in France^13–16^.

### Type of study

As this was a new topic, we used an exploratory sequential mixed methods approach that combined the collection and analysis of quantitative and qualitative data. The first step of this approach was to collect and analyze qualitative data. Based on qualitative findings, we develop in a second step a survey to collect data for the quantitative study^17^. This methodology allows for an in-depth analysis of the topic. The Good Reporting of A Mixed Methods Study (GRAMMS) Checklist was used to ensure accurate reporting^18^.

### Qualitative data

#### Study population

We included 45 nephrologists (23 men and 22 women) from three French regions: Bretagne, Île-De-France, and Normandie. We selected the nephrologists based on their gender and years of experience to ensure maximum heterogeneity.

### Data collection

We collected data in semi-structured interviews conducted face-to-face during a scheduled appointment. ALK and three clinical research associates with experience in qualitative data collection and analysis carried out the interviews. A comprehensive, pre-tested interview guide was used to collect data. The guide included one main question “What do you think of the HAS guidelines for nephrology in your practice?”. The guide included follow-up questions, particularly on nephrologists’ perceptions of the 2015 guidelines in relation to the registration of patients on the kidney transplant waiting list. We recorded and transcribed all interviews. We drew up a summary sheet at the end of each interview.

### Data analysis

ALK et SB coded separately the collected material using the Nvivo software. We carried out a thematic content analysis using an inductive approach to capture the major themes that emerged from our corpus. We organized collective sessions with all study team members to discuss the codes in order to ensure the validity and coherence of the various codes and themes identified. Saturation was reached when the analysis did not bring any new additional theme. We used the COnsolidated criteria for REporting Qualitative research (COREQ) tool to ensure compliance with the main methodological elements that are essential in a qualitative study^19^.

#### Quantitative data analysis and data integration

Based on the specific main themes and sub-themes revealed by the qualitative data analysis, we developed the quantitative study to assess the generalizability of our results.

### Study type and population

We conducted a descriptive cross-sectional survey of nephrologists practicing in France.

### Data collection

We collected data through a questionnaire that was digitized on KoBoToolbox (a data collection, management, and visualization platform) and made available on the website of the Société Francophone de Néphrologie Dialyse et Transplantation (SFNDT; French-speaking Society of Nephrology, Dialysis and Transplantation) from October 25, 2024 to January 31, 2025^20^. The information about the questionnaire was distributed via the newsletter of the SFNDT.

### Data analysis and integration

We extracted the questionnaire data from the KoBoToolbox platform and analyzed them using the RStudio software. We presented categorical variables as numbers and percentages and compared them with the Fisher’s exact test. We set the significance level at 5%.

The integration involved using the qualitative analysis results to build the quantitative questionnaire. Then, we discussed the qualitative and quantitative results in the Discussion section to determine the extent to which the quantitative results allowed us to understand and/or generalize the qualitative findings.

### Ethical considerations

Data collection, data analyses and all methods were carried out in accordance with relevant guidelines and regulations. The experimental protocol was approved “Ecole des Hautes Etudes en Santé Publique” licensing committee. All nephrologists received an information leaflet beforehand and gave their informed consent before each interview.

## Results

### Qualitative results

The analysis of the interviews identified three main themes: nephrologists’ knowledge and sources of information on the HAS guidelines in nephrology; their perception of the HAS guidelines in nephrology and their relevance for medical practice; age limit to access the kidney transplant waiting list in the 2015 HAS guidelines. Table 1 presents a selection of quotes to illustrate these themes.

**Table 1:** Selection of quotes to illustrate the themes identified in the qualitative data analysis.

Themes	Examples of quotes
**Theme 1**: Nephrologists’ knowledge and sources of information on the HAS guidelines in nephrology	**Guidelines known, but only partially read** *"I don’t think I’m necessarily very up to date on the guidelines." (nephrologist 8)* *"So, I'll be honest, I haven’t read the latest ones, I'd been involved in writing them, well proofreading and writing, but I haven’t read the final version." (nephrologist 6)*
	**Source of information for nephrologists** *"I don’t receive emails from the HAS at my nephrologist’s address to tell me: 'watch out, there’s an alert, a new piece of information’." (nephrologist 14)* *"Most of the time, it’s the SFNDT website that tells us: 'Hey, there’s a report on the renal biopsy role that’s been published…'. Yes, that’s often where we get our information…" (nephrologist 2)*
**Theme 2**: Nephrologists’ perception of the HAS guidelines in nephrology and their relevance for medical practice	**Perception of the HAS guidelines in nephrology** *"I like the guidelines, because it allows us to see if we’re not deviating from what’s being done in general. " (nephrologist 15)* *"It’s not that I don’t agree with the guidelines, it’s just that it’s a guide on a piece of paper, but I don’t have… I'm not looking at paper, I'm looking at a patient." (nephrologist 12)*
	**Relevance of the HAS guidelines for medical practice** *"Yes, it [the guidelines] shows a perfectly logical theoretical process. But in practice, it’s not always relevant. Well, it’s not always clear, I'm thinking about the monitoring of patients with kidney failure." (nephrologist 16)*
**Theme 3**: Age limit to access the kidney transplant waiting list in the 2015 HAS guidelines	*"Finally, we’ve had a lot of experience with older transplant patients who were doing much better on dialysis than after the transplant." (nephrologist 9)* *"Well, there’s the age… well, what poses the biggest problem and debate is the age limit of 85 years, where in fact there are a lot of patients who are 80, and in fact they’re so frail, it seems tricky." (nephrologist 4)* *"So, we should increase the number of medical staff to absorb the extra workload this will generate, which hasn’t been the case. So, there you are, it’s good for the patient, but it puts a strain on the medical and paramedical teams." (nephrologist 10)* *"Yes, because before we didn’t think about offering transplants to 80-year-old people, but now we’re thinking, we’re thinking about it." (nephrologist 9)*

- **Theme 1: Nephrologists’ knowledge and sources of information on the HAS guidelines in nephrology**

#### Guidelines known, but only partially read

The assessment of nephrologists’ perceptions of the HAS guidelines was based on the assumption that these guidelines are known and/or have been consulted at least once by the concerned professionals. The interviews revealed that all interviewed nephrologists were aware of the guidelines. However, their knowledge often remained general and sometimes obsolete. Most practitioners said that they had not consulted them recently, as illustrated by the following statement: *"I haven’t reviewed them recently, so I can’t tell you. I think they’re clear" (nephrologist 1).* This highlights the importance of professional intelligence and also the constraints that limit its application. Lack of time seemed to be a major obstacle to the regular updating of knowledge: *"What we don’t have is time to read pages and pages and pages of…".* (*nephrologist 2*).

#### Source of information for nephrologists

Nephrologists mentioned various sources of information about the HAS guidelines. Some consulted the HAS website directly, while others sought information from their peers or used more or less specialized social networks. For example, a nephrologist stated: "*It’s word-of-mouth between colleagues (…), or when you type ‘guideline’ on the net" (nephrologist 3),* and another said: *"I looked on the internet, on the HAS website, and then on social networks." (nephrologist 4).* Nephrologists also deplored the absence of official notifications of updates*: "We don’t get an email from the HAS saying ‘there’s a new guideline out’, so no, if I discover that there are new guidelines, I warn my colleagues" (nephrologist 3).* The lack of an official channel for disseminating information contributes to the implementation of informal and individual strategies where physicians try to gather information using the resources they consider most accessible.

However, most nephrologists also mentioned the use of more formal sources to update their knowledge, particularly specific professional channels dedicated to their specialty: *"Already through the SFNDT, where we get the information that there are new guidelines. The nephrologists’ society communicates" (nephrologist 5).*

- **Theme 2: Nephrologists’ perception of the HAS guidelines in nephrology and their relevance to medical practice**

#### Perception of the HAS guidelines in nephrology

The nephrologists’ perceptions of the HAS guidelines were ambivalent. A number of nephrologists had a rather positive view of them and thought that they were rather “clear” and useful for their professional practice. They said that they provided a frame of reference to guide their practices: "*It’s not bad, they’re quite clear and rather close to what seems to be the right thing to do". (nephrologist 6); "I find that the 2015 pre-transplant guidelines were quite clear (nephrologist 7)".*

However, sometimes, these recommendations were perceived more critically, as a tool that deprives them of their professional autonomy, and therefore of their expertise as practitioners in deciding what "should be done" for their patients in function of their clinical conditions: "*But there’s a slightly prescriptive side, I think, that’s a bit scary. It can give the impression that in the future, we won’t be able to decide how we treat our patients." (nephrologist 8).* Others said that these guidelines were difficult to read, "*sometimes a bit dense, anyway." (nephrologist 2),* or even influenced by the patient associations’ views. A nephrologist declared: *"And once again, it’s the pressure from associations to… and from the civil life of the community to say that people shouldn’t be dialyzed, that dialysis is a prison and that they should get a transplant. I have people who underwent transplantation, and they would have been better off staying on dialysis" (nephrologist 9).*

#### Relevance of the HAS guidelines for medical practice

The previous findings suggest a contrasting relevance of the HAS guidelines in the nephrologists’ practice. Some nephrologists felt that they were important and legitimate because they enabled them to readjust their practice: *"It prevents you from doing anything on your own when you’re not very well trained" (nephrologist 10).* They provided a reassuring and structuring framework for standardizing practices, but also for improving the care quality and facilitating clinical decision-making. However, their implementation also raised a number of challenges, such as access to other healthcare professionals/teams: "*It’s true that the guidelines are good, but afterwards we need to see how accessible a transplant team is, and how accessible the complementary exams that may be requested are" (nephrologist 11).* From this perspective, some nephrologists considered that the HAS guidelines were not easy to apply because they were not in line with the local clinical constraints: "*We sometimes have the impression that these guidelines are written by people who are in university hospitals, with all the exams and consultants and technical platforms 100 m away, and easy to access" (nephrologist 3).* Therefore, these guidelines could be useful for "non-specialists", like general practitioners, but not necessarily for nephrologists, especially those with years of experience who are confident on what must be done. *"For me, guidelines are a good way of guiding a non-specialist, of saying, 'Oh, well, yes, they are doing this, they are doing that, that’s that, that’s that’. But we do this… well, I don’t know, after 30 years of practice" (nephrologist 12).*

- **Theme 3: Age limit to access to the kidney transplant waiting list in the 2015 HAS guidelines**

In the 2015 guidelines to harmonize nephrologists’ practice on waiting list registration, the age limit was a salient point in the interviews. The age limit of 85 years was perceived as too high, reflecting concerns about the real benefits of transplantation versus dialysis after a certain age: *"The latest guidelines, which say that patients should be assessed up to the age of 84, I think that’s starting to sound a bit old" (nephrologist 9); "I'm a bit divided on this age limit, because in my practice, there are already many more people on the waiting list than there are grafts. So there you have it. And… [short pause] personally, I don’t think I’ve ever seen a transplant in a patient older than 80 that went well." (nephrologist 13).*

Moreover, several nephrologists mentioned the negative repercussions that the HAS guidelines had on the management of patients eligible for transplantation. Particularly, the interviews revealed that the increase in the age limit had the effect of increasing the number of patients referred by dialysis nephrologists to transplant specialists. This, in turn, generated a work overload for the transplant specialists because their numbers remained stable: *"More requests, fewer filters from dialysis specialists and a much greater mental load for transplant specialists, the people who register". (nephrologist 10).*

However, the guidelines to increase the age limit had not only negative effects. They also led to question the age limit as a discriminating criterion that helped to initiate a process of reflection on the possibility of transplantation in an older patient. For instance, one nephrologist said: *"It’s about questioning the possibility of transplantation for older adults. For a long time, I think age was a barrier, a limit of 70, and then when they freed things up by saying: 'Well, no, we can transplant people aged 80–85 if they’re in good cardiac and vascular conditions. If they have good autonomy, why not transplant them?’” (nephrologist 7).* These elements reflect an ethical and pragmatic questioning on how to optimize the allocation of kidney transplantation resources.

### Quantitative results

A total of 126 nephrologists were included in the quantitative study: 47.6% of them were women, 29.4% were younger than 41 years, 34.9% were aged between 41 and 50 years, and 33.3% were older than 50 years. The nephrologists had a median of 16 years (min–max: 1–44 years) of practice since completing their specialization residency IQR [10; 25] (Table 2).

**Table 2:** Nephrologists’ characteristics, knowledge and sources of information on the “Haute Autorité de Santé” (HAS) guidelines (quantitative study).

Features	N (126)
Gender	
Women	60 (47.6%)
Men	64 (50.8%)
Missing	2 (1.6%)
Age range	
41–50 years	44 (34.9%)
> 50 years	42 (33.3%)
≤ 40 years	37 (29.4%)
Missing	3 (2.4%)
Transplant nephrologist	
No	88 (69.8%)
Yes	32 (25.4%)
Missing	6 (4.8%)
Number of years since specialization residency	
Mean (SD)	17.70 (9.71)
Median (Q1, Q3)	16.0 (10.0, 25.0)
*Awareness/knowledge of the HAS guidelines*	
Have you ever heard about the HAS guidelines in nephrology? (N = 126)	
No	15 (11.9%)
Yes	106 (84.1%)
Missing	5 (4.0%)
Did you read the 2015 HAS guidelines on the access to the kidney transplant waiting list? (N = 126)	
No	40 (31.7%)
Yes	66 (52.4%)
Missing	20 (15.9%)
How often do you consult the HAS guidelines for a specific need? (N = 106)	
Once every two or three years	16 (15.1%)
Once per year	21 (19.8%)
Once per month	10 (9.4%)
Once per week	2 (1.9%)
Once every 3 months	23 (21.7%)
Once every 6 months	16 (15.1%)
Only once since their publication	8 (7.6%)
Never	10 (9.4%)
Do you think you are up to date with all HAS guidelines in nephrology? (n = 106)	
No	68 (64.1%)
Yes	36 (34.0%)
Missing	2 (1.9%)
*Source of information on the HAS guidelines (N = 106)*	
HAS website	
No	33 (31.1%)
Yes	73 (68.9%)
Emails from SFNDT	
No	35 (33.0%)
Yes	71 (67.0%)
Conferences	
No	45 (42.5%)
Yes	61 (57.5%)
Discussions with colleagues, staff	
No	37 (34.9%)
Yes	69 (65.1%)
Other sources	
No	103 (97.2%)
Yes	3 (2.8%)
Social networks	
No	99 (93.4%)
Yes	7 (6.6%)
Being part of the editorial group	
No	101 (95.3%)
Yes	5 (4.7%)
*Perception of the HAS guidelines and their relevance to medical practice (N = 106)*	
Clear guidelines	
Disagree	5 (4.7%)
Neither agree nor disagree	9 (8.5%)
Agree	91 (85.8%)
Missing	1 (0.9%)
Complete guidelines	
Disagree	11 (10.4%)
Neither agree nor disagree	9 (8.5%)
Agree	85 (80.2%)
Missing	1 (0.9%)
Unsuitable guidelines	
Disagree	61 (57.5%)
Neither agree nor disagree	22 (20.8%)
Agree	22 (20.8%)
Missing	1 (0.9%)
Guidelines perceived as a constraint	
Disagree	79 (74.5%)
Neither agree nor disagree	15 (14.2%)
Agree	11 (10.4%)
Missing	1 (0.9%)
Regularly updated guidelines	
Disagree	47 (44.3%)
Neither agree nor disagree	24 (22.6%)
Agree	34 (32.1%)
Missing	1 (0.9%)
Too long guidelines	
Disagree	36 (34.0%)
Neither agree nor disagree	23 (21.7%)
Agree	45 (42.5%)
Missing	2 (1.9%)
Real life-based guidelines	
Disagree	34 (32.1%)
Neither agree nor disagree	15 (14.2%)
Agree	53 (50.0%)
Missing	4 (3.8%)
Too much top-down distribution of the guidelines	
Disagree	20 (18.9%)
Neither agree nor disagree	44 (41.5%)
Agree	38 (35.8%)
Missing	4 (3.8%)
Guidelines to be made freely available for feedback before publication	
Disagree	19 (17.9%)
Neither agree nor disagree	17 (16.0%)
Agree	67 (63.2%)
Missing	3 (2.8%)
Useful guidelines for medical practice	
No	9 (8.49%)
Yes	84 (79.25%)
Don’t know	13 (12.26%)
Guidelines consulted in case of doubt	
No	22 (20.75%)
Yes	83 (78.30%)
Don’t know	1 (0.94%)
*Impact of the 2015 HAS guidelines*	
Positive impact on medical practice (N = 106)	
No	44 (41.5%)
Yes	23 (21.7%)
Don’t know	39 (36.8%)
Increase in the number of referred patients (N = 23)	
No	11 (47.8%)
Yes	12 (52.2%)
Improved access to the kidney transplant lists (N = 23)	
No	11 (47.8%)
Yes	12 (52.2%)
Improved team awareness of the transplant project (N = 23)	
No	13 (56.5%)
Yes	10 (43.5%)
Other impacts (N = 23)	
No	22 (95.6%)
Yes	1 (4.4%)
Negative impact of the 2015 HAS guidelines (N = 106)	
Don’t know	33 (31.1%)
No	61 (57.5%)
Yes	12 (11.3%)
Increased workload (N = 12)	
No	7 (58.3%)
Yes	5 (41.7%)
Increased mental workload (N = 12)	
No	9 (75%)
Yes	3 (25%)
Increased number of referred patients (N = 12)	
No	9 (75%)
Yes	3 (25%)
Longer waiting times for appointments (N = 12)	
No	6 (50%)
Yes	6 (50%)
Longer waiting times for patients on the kidney transplant list (N = 12)	
No	10 (83.3%)
Yes	2 (16.7%)

**Knowledge and sources of information on the HAS guidelines in nephrology **(Table 2)

Overall, 106/126 nephrologists (84.1%) had already heard of the HAS guidelines in nephrology: 21.7% of them (23/106) consulted them at least once every three months, 15.1% (16/106) once every six months, and 19.8% (21/106) once per year. Therefore, regular reading of these guidelines was rare: 9.4% (10/106) read them once per month, 9.4% (10/106) never read them, and 7.6% (8/106) had read them only once since their publication. Moreover, 36/106 (34%) felt that they were up to date with the guidelines in nephrology and 66/106 nephrologists (52.4%) declared that they had read the 2015 HAS guidelines on the patients’ registration in the kidney transplant waiting list.

There was no significant association between being up to date with the guidelines and the nephrologists’ gender (p = 0.54) and length of practice (p = 0.84).

Concerning the sources of information on nephrology-related guidelines (n = 106 interviewees), 68.9% of nephrologists said they were mainly informed via the HAS website, 67% via SFNDT emails, 65.1% through informal discussions between colleagues, 57.5% at congresses, and 6.6% via social networks. Five nephrologists (4.7%) said they had been informed because they were part of the group that drafted the recommendations.

**Perception of the HAS guidelines in nephrology and their relevance for medical practice** (Table 2)

Concerning this point, 85.8% and 80.2% of the 106 nephrologists who had already heard of them found that they were “clear” and “complete”, respectively. Moreover, 79.2% of them found that the guidelines in general were useful for their medical practice, and 78.3% consulted them in case of doubt, to better manage their patients. The perceived usefulness of the guidelines was not associated with the nephrologists’ number of years of practice (p = 0.88) or gender (p = 0.14). However, 20.8% of the 106 nephrologists felt that the guidelines were inappropriate, and 10.4% that they represented a constraint on their practice. Moreover, 42.5% found them too long and 32.1% disagreed with the statement that the HAS guidelines take sufficiently into account the realities in the field (50% agreed and 14.2% did not have an opinion). Furthermore, 35.8% of nephrologists found the distribution method too top-down.

Regarding the 2015 guidelines on access to the kidney transplant waiting list, 57/106 nephrologists (53.8%) found them useful to reduce disparities in the access to kidney transplantation, and 29/106 nephrologists (27.04%) considered them necessary to reduce disparities between men and women in the access to the transplant waiting list. In addition, 29/106 nephrologists found that they were useful for regional health agency/hospital directors and 49/106 for patients. Lastly, 28/106 (26.4%) nephrologists felt that these guidelines had taken into account the views of patient associations.

Moreover, 23/106 (21.70%) nephrologists thought that they had a positive impact on their practice. Specifically, 12/23 (52.17%) believed that they had led to an increase in the number of patients referred and had improved access to the waiting list, and 10/23 (43.48%) thought that they had improved the team awareness of the transplant project.

Conversely, 12/106 nephrologists (11.32%) believed that the 2015 guidelines had negative repercussions, including an increase of their workload (5/12; 41.7%), of the number of patients referred (3/12; 25%), of their mental workload (3/12; 25%), of the waiting time for an appointment (50%), and of the waiting time on the transplant waiting list for patients (2/12; 16.7%). There was a significant association between the nephrologist type (transplant or non-transplant) and the perceived negative impact of the 2015 guidelines (p = 0.023). Indeed, 6/26 transplant nephrologists and 3/75 non-transplant nephrologists thought that these guidelines had a negative impact on the access to the waiting list.

**Age limit to access the renal transplant waiting list in the 2015 HAS guidelines** (Table 3)

**Table 3:** Nephrologists perceptions on the 2015 Haute Autorité de Santé (HAS) guidelines on the access to kidney transplantation waiting list and their perception of the age limit.

The age limit of 85 years is reasonable (N = 106)	
No	46 (43.4%)
Yes	39 (36.8%)
Don’t know	21 (19.8%)
What would be your ideal age limit? (N = 46)	
75 years	13 (28.3%)
80 years	26 (56.5%)
90 years	1 (2.1%)
Missing	6 (13.1%)
Age limit of 85 years not reasonable: patients’ clinical status (N = 46)	
No	9 (19.6%)
Yes	37 (80.4%)
Age limit of 85 years not reasonable: transplant outcome (N = 46)	
No	23 (50%)
Yes	23 (50%)
Age limit of 85 years not reasonable: surgery and anesthesia risks (N = 46)	
No	21 (45.7%)
Yes	25 (54.3%)
Age limit of 85 years not reasonable: post-transplant compliance (N = 46)	
No	44 (95.7%)
Yes	2 (4.3%)
Age limit of 85 years not reasonable: organ shortage (N = 46)	
No	25 (54.3%)
Yes	21 (45.7%)
Age limit of 85 years not reasonable: other reasons (N = 46)	
No	40 (87%)
Yes	6 (13%)
The 2015 HAS guidelines are needed to reduce disparities in the access to the transplant waiting list (N = 106)	
No	13 (12.3%)
Yes	57 (53.8%)
Don’t know	36 (34.0%)
The 2015 HAS guidelines are needed to reduce disparities between men and women (N = 106)	
No	36 (34.0%)
Yes	29 (27.4%)
Don’t know	39 (36.8%)
Missing	2 (1.9%)
The 2015 HAS guidelines are useful for regional health agency and/or hospital directors (N = 106)	
No	13 (12.3%)
Yes	29 (27.4%)
Don’t know	60 (56.6%)
Missing	4 (3.8%)
The 2015 HAS guidelines are useful for patients (N = 106)	
No	13 (12.3%)
Yes	49 (46.2%)
Don’t know	44 (41.5%)
Do you think that the 2015 HAS guidelines took into account the patient associations’ views? (N = 106)	
No	5 (4.7%)
Yes	28 (26.4%)
Don’t know	16 (15.1%)
Missing	57 (53.8%)

To the question about the age limit of 85 years for referring a patient to a transplant team, 46/106 nephrologists (43.4%) answered that this threshold was unreasonable (i.e. too old). Their reasons were related to the patients’ clinical condition (37/46 nephrologists), transplant outcomes (23/46 nephrologists), surgery/anesthesia risks for these patients (25/46 nephrologists), post-transplant compliance (2/46 nephrologists), and organ shortage (21/46). To the question of the ideal age limit, 26 nephrologists proposed 80 years and 13 nephrologists proposed 75 years.

## Discussion

The aim of this study was to understand how nephrologists perceive the HAS guidelines related to their medical practice. Overall, the results of the quantitative study, which was based on the results of the qualitative study, confirmed the nephrologists’ views reported in the qualitative study. Most nephrologists were familiar with the HAS guidelines established 10 years ago, but not necessarily with their updates. The perception of the guidelines varied. Some nephrologists considered them useful because they provided a framework to guide practice, but others perceived them as unsuitable or even restrictive. To our knowledge, this is the first study in France to examine the nephrologists’ perceptions of the HAS guidelines. The sequential mixed methodological approach allowed us to explore this subject in greater depth.

Most nephrologists (84.1%) had already heard about the HAS guidelines, but they did not always had the opportunity to update their knowledge. Some nephrologists cited a lack of time that limited their ability to systematically read new recommendations, although these are essential for optimizing patient care. This qualitative result was confirmed by the quantitative study. Importantly, 42.5% of nephrologists found the guidelines “too long”. To facilitate their regular consultation, it would be useful to increase the visibility of formal channels within medical networks, to encourage their adoption through awareness-raising and clarification initiatives, and to promote their integration into the nephrologists’ daily practice. As most nephrologists stated that they obtained information mainly from the HAS website, it could be useful, for example, to send an email to them when new guidelines are issued or old ones updated. This is particularly relevant because HAS is exploring ways of updating guidelines, drawing on the lessons learned from the COVID-19 pandemic with "real-time updates"^21^. Finally, we hope that the continuing professional development programs supported by the nephrology community, particularly those dedicated to kidney transplantation, will contribute to the dissemination of guidelines^22^.

Some nephrologists had a negative perception of the HAS guidelines: 20.8% found them unsuitable and 10.4% perceived them as a constraint on their practice. Studies carried out among cardiologists on guidelines concerning refractory hypertension^23^ and dyslipidemia management^24^ showed similar trends. Indeed, 6.8% of cardiologists felt that some recommended techniques did not guarantee optimal patient safety. Our results underline the need of a more participative approach in the guideline development, with greater integration of the feedback from practitioners in the field. The implementation of co-construction mechanisms that directly involve the specialists concerned and the development of synthesis tools and interactive training could improve the adherence and applicability of guidelines in daily practice^23^. For 63.2% of nephrologists, it was crucial to gather the opinions of professionals in the field by allowing them to consult the guidelines before their publication. This underlines the need to consider the feedback from the field when updating the guidelines.

The analysis of the questionnaire answers showed that 79.2% of nephrologists felt that the HAS guidelines were useful for medical practice and 78.3% consulted them when in doubt to better manage their patients. These quantitative results are broadly in line with the qualitative findings. Moreover, no association was found between seniority of practice and perception of the usefulness of the HAS guidelines. A systematic review on the impact of clinical guidelines showed that they improved the healthcare professionals’ practice, although the effect size was very heterogeneous^25^. Clear guidelines can enhance the quality and efficiency of patients’ care and also optimize the practitioners’ diagnostic and therapeutic decisions.

However, we would like to add few nuances to these general results by recalling, for example, that 11.32% of nephrologists thought that the 2015 clinical practice guidelines on access to the waiting list had negative repercussions in terms of increased workload (41.7%) and higher number of patients referred for inclusion in the list (25%).

The question of the age limit for not referring patients to a transplant team was also addressed in this study. The qualitative data analysis suggests that this age limit was considered too high, a finding partly confirmed by the quantitative data. Indeed, 43.4% of nephrologists considered the age limit of 85 years too high to refer a patient for transplantation (in terms of risks to the patient undergoing such an operation). This underlines the dilemma that nephrologists may perceive between following the recommendations "to the letter" and relying on their professional experience.

A key element to consider is the patient’s place in the development of guidelines. According to the data collected in this study, only 26.4% of nephrologists thought that these recommendations incorporated the views of patient associations, underlining the importance they attach to taking into account the patients’ expectations and needs for improving the quality of care. This process must be rigorous, transparent and free from any conflict of interest to guarantee the guideline objectivity and maintain their central role in optimizing nephrology care^26–28^.

Despite the originality of this work, few limitations should be noted. The quantitative data were collected online and participation was voluntary so a selection bias cannot be excluded. However, our study population was fairly heterogeneous because it included male and female nephrologists of all ages, practicing in different French departments, and with or without transplantation activity. The small number of nephrologists in the quantitative study does not allow us to generalize these results to all nephrologists and outside France. However, they provide a basis from which to develop this topic. Lastly, a social desirability bias cannot be excluded in this study.

## Conclusion

This study shows how nephrologists perceive the HAS guidelines. The results will feed discussions on how to make these guidelines more accessible to nephrologists. New studies could be carried out to better quantify and qualify the effect of these guidelines on the patients’ management and on the nephrologists’ practice.

## Data Availability

The datasets used and analyzed during the current study are available from the corresponding author on reasonable request.
